# Ceftazidime-Avibactam Plus Aztreonam for the Treatment of Blood Stream Infection Caused by *Klebsiella pneumoniae* Resistant to All Beta-Lactame/Beta-Lactamase Inhibitor Combinations

**DOI:** 10.3390/antibiotics14080806

**Published:** 2025-08-07

**Authors:** Konstantinos Mantzarlis, Efstratios Manoulakas, Dimitrios Papadopoulos, Konstantina Katseli, Athanasia Makrygianni, Vassiliki Leontopoulou, Periklis Katsiafylloudis, Stelios Xitsas, Panagiotis Papamichalis, Achilleas Chovas, Demosthenes Makris, George Dimopoulos

**Affiliations:** 1 Department of Critical Care, University Hospital of Larissa, School of Medicine, University of Thessaly, 41110 Larissa, Greece; stratosfox@hotmail.com (E.M.); katselikonstantina8@gmail.com (K.K.); athama1001@hotmail.com (A.M.); vasoula_leontop@yahoo.com (V.L.); appollon7@hotmail.com (D.M.); 2 Department of Critical Care, General Hospital of Larissa, 41221 Larissa, Greece; dcpapadopoulosmd@gmail.com (D.P.); katper76@gmail.com (P.K.); ppapamich@med.uth.gr (P.P.); achovas@yahoo.de (A.C.); 3 Department of Microbiology, University Hospital of Larissa, School of Medicine, University of Thessaly, 41110 Larissa, Greece; stexit007@gmail.com; 4 Third Department of Critical Care Medicine, Medical School, National and Kapodistrian University of Athens, 11527 Athens, Greece; gdimop@med.uoa.gr

**Keywords:** pan-drug resistant *K. pneumonia*, ceftazidime-avibactam + aztreonam treatment, double carbapenem therapy, blood stream infection, survival

## Abstract

**Introduction:** The combination of ceftazidime−avibactam (CAZ-AVI) with aztreonam (ATM) may be an option for the treatment of infections due to metallo-β-lactamases (MBLs) producing bacteria, as recommended by current guidelines. MBLs protect the pathogen from any available β-lactam/β-lactamase inhibitor (BL/BLI). Moreover, in vitro and clinical data suggest that double carbapenem therapy (DCT) may be an option for such infections. **Materials and Methods:** This retrospective study was conducted in two mixed intensive care units (ICUs) at the University Hospital of Larissa, Thessaly, Greece, and the General Hospital of Larissa, Thessaly, Greece, during a three-year period (2022−2024). Mechanically ventilated patients with bloodstream infection (BSI) caused by *K. pneumoniae* resistant to all BL/BLI combinations were studied. Patients were divided into three groups: in the first, patients were treated with CAZ-AVI + ATM; in the second, with DCT; and in the third, with antibiotics other than BL/BLIs that presented in vitro susceptibility. The primary outcome of the study was the change in Sequential Organ Failure Assessment (SOFA) score between the onset of infection and the fourth day of antibiotic treatment. Secondary outcomes were SOFA score evolution during the treatment period, total duration of mechanical ventilation (MV), ICU length of stay (LOS), and ICU mortality. **Results:** A total of 95 patients were recruited. Among them, 23 patients received CAZ-AVI + AZT, 22 received DCT, and 50 patients received another antibiotic regimen which was in vitro active against the pathogen. The baseline characteristics were similar. The mean (SE) overall age was 63.2 (1.3) years. Mean (SE) Acute Physiology and Chronic Health Evaluation II (APACHE II) and SOFA scores were 16.3 (0.6) and 7.6 (0.3), respectively. The Charlson Index was similar between groups. The control group presented a statistically lower SOFA score on day 4 compared to the other two groups [mean (SE) 8.9 (1) vs. 7.4 (0.9) vs. 6.4 (0.5) for CAZ-AVI + ATM, DCT and control group, respectively (*p* = 0.045)]. The duration of mechanical ventilation, ICU LOS, and mortality were similar between the groups (*p* > 0.05). Comparison between survivors and non-survivors revealed that survivors had a lower SOFA score on the day of BSI, higher PaO_2_/FiO_2_ ratio, higher platelet counts, and lower lactate levels (*p* < 0.05). Septic shock was more frequent among non-survivors (60.3%) in comparison to survivors (27%) (*p* = 0.0015). Independent factors for mortality were PaO_2_/FiO_2_ ratio and lactate levels (*p* < 0.05). None of the antibiotic regimens received by the patients was independently associated with survival. **Conclusions:** Treatment with CAZ-AVI + ATM or DCT may offer similar clinical outcomes for patients suffering from BSI caused by *K. pneumoniae* strains resistant to all available BL/BLIs. However, larger studies are required to confirm the findings.

## 1. Introduction

Gram-negative bacteria (GNB) infections constitute a considerable percentage of intensive care unit (ICU) infections, and are associated with significant morbidity and mortality [1]. At the same time, the emergence of antibiotic resistance has become a major concern in critical care [2]. Carbapenemase-producing *Enterobacterales* (CPE) represent one of the most important threats in the field [3]. *Klebsiella pneumoniae* resistant to carbapenems (CR-KP), due to the production of *Klebsiella pneumoniae* carbapenemase (KPC), has been described as the most common pathogen worldwide [4,5]. Moreover, in recent years, the spread of metallo-β-lactamases (MBLs) has protected pathogens from the new β-lactamase inhibitors—avibactam, vaborbactam, and relebactam—increasing mortality among patients presenting bloodstream infections (BSIs) [6].

In vitro data support the use of a combination of aztreonam (ATM) with ceftazidime-avibactam (CAZ-AVI) as a treatment option for such infections [7,8]. When the combination of CAZ-AVI is insufficient to counter the pathogen’s mechanisms of resistance, a third antibiotic—ATM—is added to restore susceptibility. MBLs cannot hydrolyze ATM. However, ATM can be hydrolyzed by other non-MBL β-lactamases, which are destroyed by avibactam. The combination of CAZ-AVI + ATM is recommended as a first-line option against MBL producing *Enterobacterales* by current guidelines [9,10]. An alternative antibiotic strategy is double-carbapenem therapy (DCT), first proposed in Greece [11]. Ertapenem, with its high affinity for the carbapenemase enzyme, serves as a suicide inhibitor to bolster high concentrations of the second carbapenem and thus achieve microbiological success [12]. The combination demonstrated bactericidal effect and clinical success. However, clinical data are lacking for both antibiotic combinations, especially DCT.

The aim of our study was to compare the effectiveness of treatment with CAZ-AVI + ATM to DCT or other active antibiotics for the treatment of BSIs in critically ill, mechanically ventilated patients due to *K. pneumoniae* resistant to the new β-lactam/β-lactamase inhibitor (BL/BLI) combinations.

## 2. Results

A total of 95 patients experienced at least one episode of BSI due to *K. pneumoniae* resistant to all BL/BLI combinations. Among them, 45 patients presented with BSI due to pan-drug resistant (PDR) *K. pneumoniae*, and were treated with either CAZ-AVI + ATM (23 patients, 24%) consisting the CAZ-AVI + ATM group, or DCT (22 patients, 23%), comprising the DCT group. The remaining 50 patients (53%) had BSI due to *K. pneumoniae* resistant to all available BL/BLIs, but susceptible to at least one other antibiotic regimen (control group). Only the first episode was considered. Table 1 presents the baseline characteristics of the patients at ICU admission. The mean (SE) overall age was 63.2 (1.3) years. Mean (SE) Acute Physiology and Chronic Health Evaluation (APACHE) II and Sequential Organ Failure Assessment (SOFA) scores were 16.3 (0.6) and 7.6 (0.3), respectively. The Charlson Index was similar between groups. All patients had received antibiotics prior to the index BSI. There were no statistically significant baseline differences between the three groups. Furthermore, no differences were observed in baseline inflammatory markers [white blood cell count (WBC), lymphocytes, and C-reactive protein (CRP)] (Table 2). Patients in the CAZ-AVI + ATM group presented with *K. pneumoniae* infection on day 22 after ICU admission, DCT group patients on day 16, and control group patients on day 18. The duration of antibiotic treatment for the BSI was 11, 13, and 16 days for CAZ-AVI + ATM, DCT, and control groups, respectively (*p* = 0.19). Here, 26 patients of the control group received gentamicin as in vitro active therapy, 17 patients received colistin, 5 patients received fosfomycin, and the remaining 2 patients received chloramphenicol.

Control group presented with a statistically lower SOFA score on day 4 in comparison to the other two groups [8.9 (1.0) vs. 7.4 (0.9) vs. 6.4 (0.5) for CAZ-AVI + ATM, DCT and control group, respectively, (*p* = 0.045)] (Table 3). Duration of mechanical ventilation (MV) [38 (5.5) vs. 24 (3.7) vs. 34 (3.2) days for CAZ-AVI + ATM, DCT and control group respectively, (*p* = 0.085)], ICU length of stay (LOS) [47.9 (7.5) vs. 30.3 (3.9) vs. 41.7 (3.9) days, (*p* = 0.11)], and mortality were similar between the groups [13 (56.5%) vs. 15 (68.2%) vs. 30 (60%) patients, (*p* = 0.69)] (Table 4). Comparison between survivors and non-survivors revealed that survivors had a lower SOFA score on the day of BSI [5.6 (0.5) vs. 8.7 (0.5) for survivors and non-survivors, respectively (*p* < 0.0001)], higher PaO_2_/FiO_2_ ratio [254.6 (16.3) vs. 158.2 (13.1), (*p* < 0.0001)], higher platelet count [249 (17) vs. 161 (16) 10^9^/L, (*p* = 0.0002)], and lower lactate levels [1.1 (0.1) vs. 3.6 (0.5) mmol/L, (*p* < 0.0001)]. Septic shock was more frequent for non-survivors (60.3%) in comparison to survivors (27%) (*p* = 0.0015) (Table 5). Independent risk factors for mortality after multivariate analysis were PaO_2_/FiO_2_ ratio [odds ratio (OR) 0.95, 95% confidence interval (CI) 0.91–0.99, (*p* = 0.019)] and lactate levels [OR 29.89, 95% CI 2.45–364.2, (*p* = 0.008)].

## 3. Discussion

In the present study, we aimed to investigate the outcomes of patients presenting with BSI due to *K. pneumoniae* resistant to all BL/BLIs combinations who were treated with two different antibiotic combinations: CAZ-AVI + ATM and DCT (more specifically, meropenem and ertapenem). The outcomes were compared with patients who were treated with active in vitro antibiotic regimens other than BL/BLI. This is the first study to consider critically ill, mechanically ventilated patients in this context. Patients who received active therapy had lower SOFA scores on day 4 of the index infection in comparison to the other two groups, but combination treatments lead to statistically similar mortality, ICU LOS, and MV duration. The SOFA score on day 7 for the control group showed a trend towards being lower, but it was not statistically different between the groups. The fact that the control group showed improvement in multi-organ failure suggests the effectiveness of treatment. On the other hand, patients who received combination therapy had higher SOFA scores in comparison to the control group, but the mortality rate was similar; moreover, the other secondary outcomes were also not statistically different. It has to be mentioned that for the CAZ-AVI + ATM group, the SOFA score was higher on day 4 and day 7, but the difference was not statistically significant. It must be underlined that the outcome of critically ill patients is multifactorial. Thus, it is not safe to conclude that the increase in SOFA score is indicative of treatment failure considering that mortality and other secondary outcomes were also similar to the other groups. In summary, combination therapy can affect the course of the infection, leading to more favorable outcomes. Finally, patients that received CAZ-AVI + ATM presented BSI later in comparison to the other two groups, but that was not statistically different.

Whether or not the combination of CAZ-AVI + ATM is effective is not well documented. The source of the data is case reports and case series [13,14,15,16,17,18]. There are only two prospective multi-center observational studies examining the effectiveness of CAZ-AVI + ATM combination therapy [19,20]. The first was conducted in several hospitals in Greece and Italy and included patients with BSI secondary to *K. pneumoniae* producing MBL [19]. The CAZ-ACI + ATM combination offered a therapeutic advantage over the other antibiotic regimens that were used, since it was associated with lower 30-day mortality [hazard ratio (HR), 0.37 and 95% confidence interval (CI), 0.13–0.74, (*p* = 0.01)], lower clinical failure at day 14 [HR, 0.30, 95% CI, 0.14–0.65, (*p* = 0.002)], and shorter length of stay [HR, 0.49, 95% CI, 0.30–0.82], (*p* = 0.007)]. The result was contrary to the second study conducted in Italy, where CAZ-AVI + ATM was associated with lower mortality in comparison to other combinations but presented similar mortality to patients who received active antibiotics other than colistin [20]. Sensitivity analysis showed that CAZ-AVI + ATM compared to colistin was independently associated with a reduced 30-day mortality rate [HR, 0.39, 95% CI, 0.18–0.86, (*p* = 0.02)]. It should be noticed that the patients included in the present study were all hospitalized in the ICU receiving MV, and only BSIs due to *K. pneumonia* were taken into account. The different study populations may explain the results between the studies.

Clinical data about the efficacy of DCT are equally scarce. Only case reports and retrospective studies with relatively small patient numbers and no control groups [21,22,23,24,25,26,27] are available. The most commonly reported infections are BSI and pneumonia. Patient outcomes are usually favorable both clinically and microbiologically, with microbiological clearance achieved during the follow-up period. In the largest study published to date [28], 48 patients treated with DCT were matched with 96 patients who received standard therapy. Although septic shock occurred more frequently in patients receiving DCT, they had lower 28-day mortality compared to the control group [29.2% vs. 47.9%, (*p* = 0.04)], and both clinical cure and microbiological eradication were significantly higher when DCT was used [65% vs. 31.3%, (*p* = 0.03) and 57.9% vs. 25.9%, (*p* = 0.04), respectively]. The DCT regimen was associated with a reduction in 28-day mortality in the logistic regression model (OR 0.33, 95% CI 0.13–0.87). In two other studies [29,30], DCT therapy was also effective compared to primarily with colistin, even if mortality was not statistically different between the groups. In a systematic review and meta-analysis of studies involving DCT therapy [31], DCT was found to be as effective as other antibiotics, with a similar treatment response. Patients who received DCT had lower mortality than comparators [OR 0.44, 95% CI 0.24–0.82, (*p* = 0.009)]. The authors concluded that DCT may represent a viable alternative therapeutic option. Our results are similar: patients treated with DCT did not have statistically different outcomes, such as mortality or ICU LOS, compared with the other regimens. Moreover, this is the first study that compares DCT therapy to CAZ-AVI + ATM for BSIs. The effects of DCT are comparable with those of CAZ-AVI + ATM; therefore, DCT therapy may constitute an alternative treatment option. Nevertheless, there remains a need for more high-quality clinical trials to address the efficacy and safety of this specific antibiotic combination.

The present study has several limitations. It was conducted in two hospitals in a specific region, and the results should be interpreted cautiously. The relatively small cohort may limit the generalizability of the findings. Furthermore, patients that received combination therapy presented infection due to PDR strains, but control group patients’ infection was caused by extensively drug-resistant (XDR) strains. Mechanisms of resistance were not investigated, and synergy testing was not available. Hetero-resistance was not studied. The production of MBL is the main mechanism of resistance for the newer BL/BLIs, but since other mechanisms of resistance may also be involved it cannot be concluded that CAZ-AVI + ATM or DCT are effective against MBL-producing *K. pneumoniae* strains. Prior antibiotics were not recorded; therefore, no potential effects on resistant mechanisms could be identified. Nevertheless, our findings may form the basis for a larger future investigation.

## 4. Materials and Methods

The study was retrospective; patients’ records from two ICUs at the University hospital of Larissa, Thessaly, Greece, and General Hospital of Larissa, Greece, were reviewed for a period of 36 months (2022–2024). For inclusion in the study, the following criteria were applied: (a) ICU admission; (b) invasive mechanical ventilation for >48 h; and (c) BSI caused by *K. pneumoniae* resistant to all BL/BLI combinations, including the newer CAZ-AVI, meropenem−vaborbactam, and imipenem−cilastatin−relebactam. Patients were excluded if: (a) they were under 18 years of age; (b) they were readmitted to the ICU; (c) they were treated with antibiotics other than CAZ-AVI + ATM or DCT for PDR strains. Only the first episode of BSI was included. Patients were then divided into three groups: those in the first group presented BSI due to PDR *K. pneumoniae* resistant to all BL/BLI combinations and any other available antibiotic regimen, and were treated with CAZ-AVI plus ATM (CAZ-AVI + ATM group); the second group consisted of patients infected with PDR *K. pneumoniae*, as the first group, and were treated with DCT (DCT group); and the third group presented BSI due to *K. pneumoniae* resistant to all BL/BLI combinations, but it was sensitive to at least one other antibiotic (XDR strain), and therefore, received effective therapy (control group). All patients that were included in the DCT group received meropenem plus ertapenem; ertapenem was administered daily 1 h prior to the first dose of meropenem.

### 4.1. Outcome

The primary outcome of the study was the evolution of multi-organ failure between the first and the fourth day of infection, as reflected by the change in SOFA score. Secondary outcomes were SOFA score evolution during treatment period, total duration of MV, ICU LOS, and ICU mortality.

### 4.2. Definitions

BSI was defined according to the Center of Disease Control (CDC) criteria [32]. PDR was the strain of *K. pneumoniae* that was non-susceptible to all agents in all antimicrobial categories. As XDR defined the strain that presented susceptibility only to one or two antimicrobial categories. The administration of in vitro active antibiotics for at least 48h was defined as appropriate therapy [33].

### 4.3. Clinical Assessment

Characteristics recorded included age, sex, illness severity (based on APACHE II and SOFA scores), medical history, history of prior antibiotic use, hospitalization, and relevant clinical and laboratory findings related to the index BSI.

### 4.4. Microbiology

The interpretation of results was based on European Committee on Antimicrobial Susceptibility Testing (EUCAST) breakpoints. The VITEK 2 automated system (bioMerieux, Marcy l’ Etoile, France) was used for pathogen identification and susceptibility testing. E-test was used for used to determine the minimum inhibitory concentration (MIC) for CAZ-AVI, meropenem−vaborbactam and imipenem−cilastatin−relebactam, and broth microdilution for colistin.

### 4.5. Statistical Analysis

Categorical variables are presented as frequencies (%) and categorical variables as means (standard error, SE). Chi-square test or Fisher’s exact test were used to compare categorical variables, and the Mann−Whitney U test, or Kruskal−Wallis test for continuous variables. The Kolmogorov−Smirnov test was used to assess data distribution normality, and the Wilcoxon test to evaluate with-group differences. Finally, stepwise logistic regression was used to select predictors for mortality. Only variables with a *p*-value < 0.05 were included in the models. Data analysis was performed using GraphPad Prism 5 (GraphPad by Dotmatics Software Development, San Diego, CA, USA, 2007) and IBM SPSS statistics v.20 for Windows.

## 5. Conclusions

In our study, CAZ-AVI + ATM and DCT present comparable effectiveness with appropriate therapy for the treatment of patients presenting with BSI caused by *K. pneumoniae* strains resistant to all available BL/BLIs including the newer CAZ-AVI, meropenem−vaborbactam and imipenem−cilastatin−relebactam. However, larger prospective studies are required to confirm the results of the present study.

## Figures and Tables

**Table 1:** Baseline characteristics.

	CAZ-AVI + ATM (*n* = 23)	DCT (*n* = 22)	Control Group (*n* = 50)	All Patients (*n* = 95)	*p*
Age (years), mean (SE)	58 (3.3)	66.8 (1.9)	64 (1.8)	63.2 (1.3)	0.06
Sex (male)	18 (78.3)	17 (77.3)	28 (56)	63 (66.3)	0.08
APACHE II score, mean (SE)	17.8 (1.5)	16.3 (1)	15.7 (0.8)	16.3 (0.6)	0.36
SOFA score, mean (SE)	8.6 (0.7)	7.5 (0.5)	7.3 (0.3)	7.6 (0.3)	0.12
Charlson Index, mean (SE)	4.2 (0.6)	3.9 (0.4)	5.4 (0.5)	4.8 (0.3)	0.08
Immunosuppression	1 (4)	0 (0)	3 (6)	4 (4)	0.50
Hospitalisation during the last 30 days (yes)	16 (69.6)	13 (59.1)	32 (64)	61 (64.2)	0.33
Antibiotics during the last 30 days (yes)	15 (65.2)	13 (59.1)	27 (54)	55 (57.9%)	0.40

Data are presented as *n* (%) of patients, unless otherwise specified; APACHE II, Acute Physiology and Chronic Health Evaluation; SOFA, Sequential Organ Failure Assessment. Results by univariate analysis.

**Table 2:** Patients’ characteristics on day 1 of BSI.

	CAZ-AVI + ATM (*n* = 23)	DCT (*n* = 22)	Control Group (*n* = 50)	All Patients (*n* = 95)	*p*
WBC (10^9^/L), mean (SE), (normal range 5–10 × 10^9^/L)	14.48 (1.26)	13.07 (1.05)	12.08 (0.8)	12.890 (0.58)	0.97
Lymphocytes (10^9^/L), mean (SE), (normal range 1–4 × 10^9^/L)	1.13 (0.16)	2.03 (0.54)	1.09 (0.12)	1.32 (0.15)	0.71
CRP (mg/dl), mean (SE), (normal range < 1.0 mg/dl)	9.8 (1.7)	6.1 (1.4)	9.1 (1.4)	8.5 (0.9)	0.89
Lactate (mmol/L), mean (SE)	3.5 (0.9)	1.4 (0.1)	1.7 (0.3)	2.1 (0.03)	0.66
Total bilirubin (mg/dl), mean (SE), (normal range 0.3–1.0 mg/dl)	1.2 (0.4)	0.5 (0.1)	0.7 (0.1)	0.8 (0.1)	0.74
Platelets (10^9^/L), mean (SE), (normal range 150–400 × 10^9^/L)	285 (31)	310 (31)	255 (14)	275 (13)	0.32
INR, mean (SE) (normal ramge 0.8–1.1)	1.4 (0.1)	1.2 (0.04)	1.4 (0.08)	1.3 (0.05)	0.63
Septic shock (yes)	11 (47.8)	15 (68.2)	19(38)	45 (47.4)	0.07
PaO_2_/FiO_2_ ratio, mean (SE)	240.8 (24.4)	144 (13.2)	197.4 (16.3)	195.5 (11.3)	0.01
Acute kidney injury (yes)	6 (26.1)	10 (45.5)	14 (28)	30 (31.6)	0.28
Hemofiltration (yes)	8 (34.8)	9 (40.9)	9 (18)	26 (27.4)	0.08
Active antibiotics within 24 h (yes)	11 (47.8)	6 (27.3)	32 (64)	49 (51.6)	0.02

Data are presented as *n* (%) of patients, unless otherwise specified; WBC, white blood cell; CRP, C-reactive protein; INR, international normalized ratio. Results by univariate analysis.

**Table 3:** SOFA score for day 0, day 4 and day 7 of BSI.

	CAZ-AVI + ATM (*n* = 23)	DCT (*n* = 22)	Control Group (*n* = 50)	*p*
Day 0	8 (0.8)	8.3 (0.7)	6.9 (0.5)	0.26
Day 4	8.9 (1)	7.4 (0.9)	6.4 (0.5)	0.045
Day 7	8.8 (1)	7.3 (1.1)	6.1 (0.6)	0.06

Data are presented as mean (SE); SOFA, Sequential Organ Failure Assessment; BSI, bloodstream infection; *p*, comparison between the three groups. Results by univariate analysis.

**Table 4:** Duration of MV, ICU stay, and mortality.

	CAZ-AVI + ATM (*n* = 23)	DCT (*n* = 22)	Control Group (*n* = 50)	*p*
MV duration (days), mean (SE)	38 (5.5)	24 (3.7)	34 (3.2)	0.085
ICU LOS (days), mean (SE)	47.9 (7.5)	30.3 (3.9)	41.7 (3.9)	0.11
Mortality (%)	13 (56.5)	15 (68.2)	30 (60)	0.69

Data are presented as *n* (%) of patients unless otherwise specified; MV, mechanical ventilation; ICU, intensive care unit; LOS, length of stay; *p*, comparison between the three groups. Results by univariate analysis.

**Table 5:** Comparison between survivors and non-survivors.

	Non-Survivors (*n* = 58)	Survivors (*n* = 37)	*p*
Age (years), mean (SE)	64.8 (1.5)	60.6 (2.5)	0.26
Sex (male)	40 (69)	23 (62.2)	0.51
APACHE II score on ICU admission, mean (SE)	16.3 (0.7)	16.3 (1)	0.62
Charlson Index, mean (SE)	5.2 (0.4)	4.1 (0.4)	0.32
Immunosuppression (yes)	4 (6.9)	0 (0)	0.15
SOFA score on day of BSI, mean (SE)	8.7 (0.5)	5.6 (0.5)	<0.0001
CAZ-AVI + ATM	13	10	0.61
DCT	15	7	0.44
Control group	30	30	0.83
Acute kidney injury (yes) on day of BSI	22 (37.9)	8 (21.6)	0.12
PaO_2_/FiO_2_ ratio on day of BSI, mean (SE)	158.2 (13.1)	254.6 (16.3)	<0.0001
Septic shock (yes) on day of BSI	35 (60.3)	10 (27)	0.0015
INR on day of BSI, mean (SE)	1.3 (0.06)	1.2 (0.03)	0.29
Platelets (10^9^/L) on day of BSI, mean (SE)	161 (16)	249 (17)	0.0002
Lactate (mmol/L) on day of BSI, mean (SE)	3.6 (0.5)	1.1 (0.1)	<0.0001

Data represent no. (%) of patients unless otherwise specified; APACHE II, Acute Physiology and Chronic Health Evaluation; ICU, intensive care unit; SOFA, Sequential Organ Failure Assessment; BSI, bloodstream infection; CAZ-AVI, ceftazidime-avibactam; ATM, aztreonam; DCT, double carbapenem therapy; INR, international normalized ratio; *p*, comparison between survivors and non-survivors. Results by univariate analysis.

## Data Availability

Data are contained within the article.
